# From the Outside Looking In

**Published:** 1913-11-15

**Authors:** Jesse A. Jarvis

**Affiliations:** Ashland, Oregon


### FROM THE OUTSIDE LOOKING IN.

BY JESSE A. JARVIS, ASHLAND, OREGON.

I belong to a family of professional men. My husband is a physician; my brother is a dentist.

For fifteen years I have been an interested listener to discussions regarding new fields of research. The science and art of dentistry and medicine and the patient as viewed from the standpoint of the practitioner. I have quite grown to share in my husband's joy over finding "a beautiful mitral murmur" and to view with interest a case of tri-facial neuralgia that has withstood treatment for an unholy length of time.

I can accept intelligently the statement that appears sardonic to the lay mind, "The operation was successful but the patient died."

It was while listening to a discussion of an operation of this latter variety that it occurred to the writer that it might not be amiss to present to the profession a short paper that would show the opposite side of the picture, namely that of the practitioner from the standpoint of the patient.

I have suffered all my life from the fragile, sensitive teeth that are the natural outcome of a nervous and delicate childhood; but some inbred abhorrence of what our country cousins call "store teeth" has led me to endure all things to preserve them to myself with the result that I have spent much of my existence in a dentist's chair.

I believe it is said with authority that our olfactory sense is the one most strong in the power of association. As you go into the average dental office what is the first thing that strikes your senses? Is it not safe to say carbolic? For myself I do not object to the odor; it is cleanly, antiseptic and not sickening, but it is not suggestive of the normal, happy things of life. Perhaps a hint of creolin is a close second, which to every lover of canines spells "dog-soap." As I belong to the class of doggy-people, again I am not greatly annoyed, but there are many women who will be actually sickened by the smell.

So used have we become, through long years of custom, and so strong is the power of anticipation that it takes more than the mere absence of these things to persuade one that they are not there.

I have in mind an office almost unique in my experience where you are greeted the minute you enter by the fragrance of flowers; in early spring you will find great bowls of pink and white lilacs; later when June brings her roses, the place is a bower of fragrant beauty. Even in winter when the hot house is the last resort, there will be spicy carnations, a few great, ragged, red blossoms in an Arts & Crafts vase against a dull green wall.

There are few people who, consciously or not, will fail to be quieted by such an influence; and relaxation will to an extent replace the high tension of anticipated suffering.

There is an office in Philadelphia with which I am very familiar. The rooms are large and sunny and filled with big easy mission chairs of the best form and finish, a large divan holds enough leather pillows to suggest a veritable cosy corner, but the thing that first attracts your eye upon entering is an open piano with a violin resting on top. This man is a really thorough musician and has continually under his guidance a succession of boys and girls, young men and women who are working hard to get on their feet in the musical world. He is too busy to give his time and attention to those who can never rise above the rank of parlor performers, but he opens his home to those who show true genius and they are welcome to come and practice in his office; not technical exercises, but rare parts of their work that will give pleasure to the listener.

The old adage, "Music hath charms to soothe the savage breast," is as true today as when it was written and it is certainly easier to endure pain when listening to Bach's Aria or the Berceuse from Jocelyn when played by a violinist of promise.

Do not understand me to mean that these agreeable and diverting surroundings ever take the place of fundamental cleanliness.

We have all, I believe, at some time experienced the shock of meeting a beautifully dressed and daintily perfumed woman and being completely disillusioned by a chance glimpse of untidy lingerie or other negligence.

At the present day every intelligent woman demands cleanliness that shall be not only comparative but absolute; whether this be inherent, or the result of education and environment, the great fact remains that she will have it and will not be satisfied with her dental work until she find a man who comes up to her requirements.

Not only is this true of the cultured class, but as we descend rung after rung of the social ladder, it is surprising to note the amount of information possessed by our humble sisters regarding germs and microbes.

Their sources of information may be dubious and some of their conclusions truly weird, but the demands they make are practically the same as those we find in higher life.

There is in one of our larger cities a young man who bids fair to be one of the "big men" of dentistry. His work is above reproach; he is cultured and attractive; his offices are the last word of good taste and comfort; his practice is drawn largely from the social people who form his social set.

A short time ago a lady who was a newcomer to the city asked me to recommend to her a dentist; she was well-to-do and had the fragile teeth that mean a steady patient. Her ten-year-old son was a subject for orthodontia. I gave her the name of the man in question, feeling sure that I had brought the right people together. A few weeks later we chanced to meet and I asked her how she had been pleased with her work. She said: "Undoubtedly he knows his business but he was so reeking with tobacco that I couldn't stand having him near me."

To the present day that young man is wondering why he lost that very desirable patient.

So closely allied to this matter of personal cleanliness that the two should go hand in hand, is that of the corresponding moral attribute. If one can say of a dentist, "he is a good man not only in his professional but private life," the practice he builds will rest upon pillars that will give it a permanent support.

Mothers do not wish to trust their young daughters to the care of a man, whatever his ability may be, whose reputation is not like that of Caesar's wife. Pass in review the career of any man who has had a long and enviable practice, or consider that of any successful practitioner of today, and you will find few exceptions to this rule, that they are men not only skillful and honest in their work, but deserving of the absolute loyalty and trust of the people who come under their care.

I do not wish to close this paper without relating an experience I passed through a few months ago that seemed to me little short of miraculous. I have suffered so many things for so many years in the effort to preserve my teeth that when one day my dentist, who is also a friend of long standing, came in to tell me that he was at last able to work on the most sensitive teeth without pain, I was frankly skeptical. So anxious was he to give me a demonstration that I hastened my regular semi-annual visit for a general inspection by a few weeks, and there as if made to order was a cavity in the most suitable possible place, one of the central incisors close to the gum. The bare pressure of the instrument against the tooth was almost unbearable and I realized afterwards that I was purposely subjected to that pain that the subsequent relief should be more apparent. It was nitrous oxid-oxygen that performed what seemed to me wizardry. The apparatus was adjusted and after a few seconds of its influence I found that while I retained consciousness I was absolutely numbed to any feeling of pain. I knew that the bur was being used, that it was cutting deep and hard, but so entire was the paralysis of sensation that I felt as if I must be under the influence of a complete anesthesia.

I was allowed to return to normal while the filling was being prepared and then a few minutes more of the nitrous oxid-oxygen and the work was done. I left the office almost dazed with the experience I had passed through and feeling as if I must tell everyone that the barrier of pain in the realm of dentistry had at least been overthrown.

I can affirm confidently that anyone having one such experience would travel a long distance before subjecting themselves again to the time-honored methods of treatment.

I am well aware that I am a member of the troublesome sex; many of us are nervous, too many of us are hysterical; we do not bear pain well and we are always looking for flies in the ointment, but such as we are, we are, and must be dealt with accordingly.

Our general attitude of mind I believe has been fairly set forth in this paper as by one who sees booth frcm the outside looking in and from the inside looking out.—*Pacific Gazette.*

